# Cytoprotective Effects of Natural Compounds against Oxidative Stress

**DOI:** 10.3390/antiox7100147

**Published:** 2018-10-20

**Authors:** Jay Mehta, Srujana Rayalam, Xinyu Wang

**Affiliations:** Department of Pharmaceutical Sciences, School of Pharmacy, Philadelphia College of Osteopathic Medicine-Georgia Campus, 625 Old Peachtree Road NW, Suwanee, GA 30024, USA; jayme@pcom.edu (J.M.); srujanara@pcom.edu (S.R.)

**Keywords:** cytoprotection, CAPE, curcumin, resveratrol, CDDO-Im, oxidative stress, cardiovascular diseases, obesity

## Abstract

Oxidative stress, an imbalance between reactive oxygen species and antioxidants, has been witnessed in pathophysiological states of many disorders. Compounds identified from natural sources have long been recognized to ameliorate oxidative stress due to their inherent antioxidant activities. Here, we summarize the cytoprotective effects and mechanisms of natural or naturally derived synthetic compounds against oxidative stress. These compounds include: caffeic acid phenethyl ester (CAPE) found in honey bee propolis, curcumin from turmeric roots, resveratrol abundant in grape, and 1-[2-cyano-3,12-dioxooleana-1,9(11)-dien-28-oyl] imidazole (CDDO-Im), a synthetic triterpenoid based on naturally occurring oleanolic acid. Cytoprotective effects of these compounds in diseases conditions like cardiovascular diseases and obesity to decrease oxidative stress are discussed.

## 1. Introduction

Chronic diseases including cardiovascular diseases, cancer, diabetes, obesity and neurodegenerative diseases have a great impact on a large number of populations [1]. One of the common characteristics among these diseases is the involvement of oxidative stress in the pathophysiological state [2]. Oxidative stress is a condition characterized by an imbalance between the production and timely removal of reactive oxygen species (ROS) [3]. It occurs when intracellular ROS are over produced from mitochondria and beyond the capacity of intracellular antioxidative defense systems to neutralize these reactive species. As a consequence, these excessive ROS cause oxidative damage to vital cellular components such as DNA, protein, and lipid membranes [4]. It is conceivable that oxidative stress is a potential therapeutic target for agents with antioxidant properties. 

**Figure 1:**
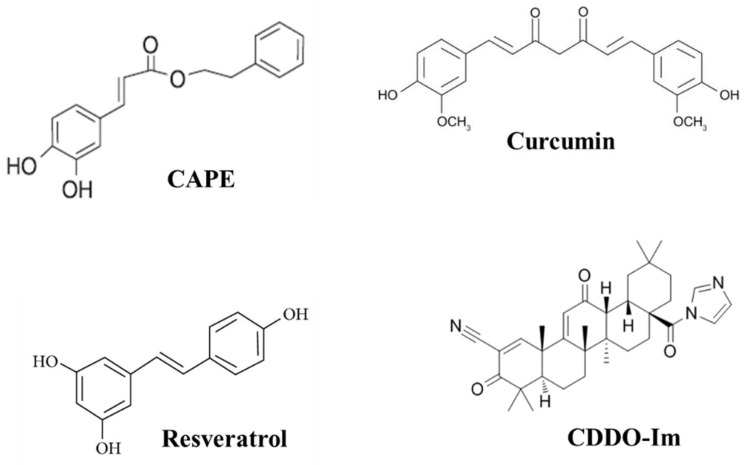
Structures of CAPE (caffeic acid phenethyl ester), Curcumin, Resveratrol, and CDDO-Im (1-[2-cyano-3,12-dioxooleana-1,9(11)-dien-28-oyl] imidazole).

Natural compounds and their derivatives have long been used for oxidative stress-involved diseases [5,6]. These bioactive molecules have raised great interest in their potential benefits largely due to their potent antioxidant activities. Here, four naturally occurring or derived compounds: caffeic acid phenethyl ester (CAPE), curcumin, resveratrol, and 1-[2-cyano-3,12-dioxooleana-1,9(11)-dien-28-oyl] imidazole (CDDO-Im) are presented, and their antioxidant effects in relation to chronic diseases is described. CAPE is found in honey bee propolis, curcumin is found in Indian spice turmeric, resveratrol is the active ingredient from grapes, and CDDO-Im is a synthetic derivative of naturally occurring triterpenoids oleanolic acid. Structures of these compounds are illustrated in Figure 1. These compounds were reported to ameliorate oxidative stress directly (free-radical scavenging) or indirectly (induction of antioxidant enzymes). However, the precise mechanism of action of these natural compounds is still largely unknown. 

## 2. Mechanism of Cytoprotection

Protection against oxidative stress occurs through two mechanisms [7]. First, the antioxidant property functions through a direct antioxidant mechanism, where the antioxidants are redox active with a short life span and are sacrificed when they act on ROS. Due to this direct effect, antioxidants need to be regenerated so that they can curtail ROS levels. The second mechanism is an indirect antioxidant effect, which can trigger self-defense mechanisms of the host cells to fight oxidative stress. Some of the established mechanisms of cytoprotection for these selected natural compounds are listed in Table 1. 

## 3. Cytoprotection of Natural Compounds

### 3.1. Caffeic Acid Phenethyl Ester

Caffeic acid phenethyl ester (CAPE), a phenolic compound, is found in the propolis of honey bees. CAPE has been reported to exhibit anti-tumor, cancer-preventative, immunomodulatory, anti-HIV, anti-oxidant, and anti-inflammatory effects. The main focus here is the antioxidant activity of CAPE, which has been demonstrated in in vitro and in vivo models [28,29]. In human umbilical vein endothelial cells (HUVECs), cellular antioxidant activity of CAPE against menadione induced oxidative stress has been reported [30]. Also, in the livers and hearts of diabetic rats, there is evidence that CAPE exerts a protective effect through the activation of antioxidant enzymes and inhibition of lipid peroxidation [29,31]. CAPE is known to induce the expression of redox-sensitive and stress-inducible protein, heme oxygenase-1 (HO-1) [9,32]. When expression of HO-1 enzyme is elevated, it has been shown to ameliorate organ dysfunction while counteracting metabolic disorders. The substrate for HO-1 is heme and heme degradation by HO-1 produces free ferrous iron, carbon monoxide and biliverdin. Increased carbon monoxide and biliverdin, which is converted to bilirubin by biliverdin reductase, can inhibit platelet aggregation, regulate vessel tone and prevent tissue injury and cell death. In addition, carbon monoxide is responsible for the anti-inflammatory effects of HO-1 [33]. Furthermore, when the redox status of thiols is challenged with radiation, oxidants, hypoxia and nitric oxide (NO), a redox imbalance occurs and ROS are overproduced leading to the activation of HO-1 to counteract this imbalance in many different types of cells [9]. The induction of HO-1 expression by CAPE to enhance cytoprotection of cells was reported through activation of the Keap1/Nrf2/ARE pathway [34]. 

It is reported that the indirect antioxidant effect of natural compounds is mediated through the activation of Keap1/Nrf2/ARE pathway resulting in the transcription and translation of phase II cytoprotective enzymes [35]. The antioxidant response element (ARE) is the regulatory region for phase II genes located upstream, the nuclear erythroid 2-related factor (Nrf2) is a transcription factor mediating both inducible and basal expression of cytoprotective enzymes, and the Kelch-like ECH-associated protein 1 (Keap1) is the target for inducers. Nrf2 is bound to Keap1 in the cytoplasm before activation and once inducers react with sulfhydryl groups of Keap1, Nrf2 is released and eventually translocated into the nucleus, where it binds to and activates ARE. ARE acts as a promoter/enhancer to regulate the genes of detoxifying and antioxidant phase II enzymes [36]. The Nrf2/ARE pathway can be activated in a Keap1-independent manner, where activation of the Nrf2 protein is mediated through a phosphorylation event which can be carried out by different signaling pathways such as phosphatidylinositol 3-kinase/protein kinase B (PI3K/Akt) and mitogen-activated protein kinase (MAPK) [37]. Kim et al., reported that the antioxidant effects exerted by CAPE may be partially mediated through ERK-Nrf2 signaling which leads to the induction of HO-1. However, further studies are needed to identify the precise molecular mechanism of action of CAPE [34]. 

### 3.2. Curcumin

Intensive studies have been performed on curcumin. This yellow pigmented compound was found in the roots of turmeric, *Curcuma long* L. and has been used in medicine for a long time in India and Southeast Asia. The mechanism of curcumin mostly occurs through Nrf2 activation and is facilitated by an electrophilic group with α,β-unsaturated carbonyl bonds as Michael reaction acceptors which interact with cysteine residues of Keap1, therefore releasing Nrf2 after the interaction [38,39]. The concentrations of curcumin required to cause the maximum activation of the Nrf2 and NAD(P)H: quinone oxidoreductase 1 (NQO1) without the induction of cytotoxicity are 5.1 µM for Nrf2 and 7.5 µM for NQO1, respectively. These concentrations of curcumin activate the protective genes and enhance resistance of the cells to oxidative stress [40]. 

In primary kidney tubular epithelial cells (NRK-52E), the effect of curcumin on the epithelial-to-mesenchymal transition (EMT), which is associated with oxidative stress and diabetic nephropathy, is studied by exposing the NRK-52E cells to a high-glucose environment and causing oxidative stress [41]. Analysis on the prevention of EMT by using curcumin revealed that levels of Nrf2 and HO-1 protein expression were significantly higher in curcumin pretreated NRK-52E cells versus the control and this expression increased in a concentration- and time-dependent manner. This finding was further supported by using siRNA to knockdown the expression of Nrf2, which prevented the expression levels of HO-1. This study showed that curcumin is a cellular antioxidant and the mechanism can partially be attributed to the activation of Nrf2 and HO-1[41]. Curcumin’s cytoprotective effect has also been confirmed in other systems. Pretreatment of primary cultures of rats’ cerebellar granule neurons with curcumin increased the expression of HO-1 and glutathione (GSH) thereby decreasing the damage caused by hemin, which is an oxidized form of heme [42]. Hemin works as a strong reactive species to induce cellular damage by reacting with peroxides to produce free-radicals and induce oxidative stress. HO-1 has the ability to decrease prooxidant heme, increase levels of the antioxidant biliverdin, and release antiapoptotic carbon monoxide [43]. The neuroprotective effects were mediated by Nrf2 translocation into the nucleus, which suggests that there is a link between the antioxidant response of curcumin and Nrf2 [42]. 

Pretreating human primary hepatocytes with curcumin attenuated cellular damage and insulin resistance induced by the exposure to glucose oxidase. Glucose oxidase increases the activities of aspartate aminotransferase (AST) and lactate dehydrogenase (LDH) resulting in the increase of ROS and depletion of GSH. Curcumin caused Nrf2 nuclear translocation in these cells partly contributing to the protective effects. Interestingly, when primary hepatocytes were treated with wortmannin, a phosphatidylinositol-3-kinase (PI3K) inhibitor, the cytoprotective property of curcumin was partially inhibited indicating that this effect may be partially mediated through PI3K [44]. The protective role of curcumin related to PI3K and Nrf2 was also seen in vascular smooth muscle cells [45]. Curcumin increased the promoter activity and mRNA levels of aldose reductase (AR), which is an important enzyme that fights against oxidative stress, in PI3K/Akt- and p38/JNK/MAPK-dependent manners.

The cytoprotective effects of curcumin have been reported not only in vitro but also in vivo. Oral administration of curcumin, at a concentration of 50 mg/kg, was shown to attenuate the effects of oxidative stress on cardiac tissue such as apoptosis, fibrosis, hypertrophy and inflammation in mice fed with a high fat diet (HFD) [46]. HFD supplementation results in hyperlipidemia and elevated free fatty acids (FFAs) that affect vascular function [47] through the augmentation of oxidative stress and ROS levels increasing the risk for cardiovascular diseases [48]. Although several genes involved in oxidative stress are regulated by Nrf2 [49], it is not clearly understood if curcumin protects hearts from HFD-induced injury through the activation of Nrf2. Zhen et al., demonstrate that the expression of Nrf2-downstream genes like HO-1 is significantly upregulated by curcumin treatment in HFD fed mice suggesting a possible role for Nrf2 activation in protection against HFD-induced oxidative stress and cardiac injury [46]. Additionally, elevated FFAs promote inflammatory responses through activation of nuclear factor-kappa B (NF-κB) in macrophages. Curcumin supplementation decreased NF-κB activity and this inhibition of NF-κB mediated pathway was associated with a decrease in the expression of pro-inflammatory cytokines including TNF-α, IL-1β and IL-6 both in vitro and in vivo [46]. In a similar study, Sprague–Dawley rats were fed with HFD to induce development of nonalcoholic steatohepatitis and curcumin was administered to determine the effects on amelioration of this syndrome developed in non-alcoholic patients. Curcumin decreased hepatic and serum contents of TNF-α, IL-6 and malondialdehyde (MDA) as well as increased the expression of HO-1, GSH, and superoxide dismutase (SOD) with greater Nrf2 expression in the nuclei of the liver cells in these animals [50]. 

These effects of curcumin on the Nrf2 pathway have also been seen in human clinical trials. In a clinical trial, patients with type II diabetes mellitus were given an oral dose of curcumin at 500 mg/day for about 15–30 days. The results of this study showed that curcumin caused an upregulation of Nrf2 regulated proteins such as NQO1 in lymphocytes, attenuation of urinary micro-albumin excretion, suppression of lipopolysaccharide levels, reduction of inflammatory markers in lymphocytes and reduced MDA levels. This study showed that curcumin acts mainly through Nrf2 but can also attenuate inflammation through other mechanisms [51]. 

### 3.3. Resveratrol

Resveratrol is known as a phytoalexin found in red grapes as well as some other plants. Resveratrol is studied for its utilities in multiple disease conditions and it is possible that some of the beneficial effects of resveratrol can be attributed to its antioxidant effects. Like curcumin and CAPE, resveratrol also induces a significant change in the expression of Nrf2 [40]. A study on primary rat hepatocytes showed that pretreatment of the hepatocytes with resveratrol attenuated the oxidative stress-induced necrosis that was caused by *tert*-butyl hydroperoxide. The protective effects of resveratrol are mediated through the antioxidant enzymes such as glutathione S-transferase (GST), glutathione peroxidase (Gpx), NQO1, catalase and SOD which are induced by increased Nrf2 mRNA levels and increased translocation of Nrf2 into the nucleus [52]. In cultured coronary arterial endothelial cells, resveratrol induced a significant increase in Nrf2 transcription in a dose-dependent manner along with significantly increased expression of NQO1, HO-1 and γ-glutamylcysteine synthetase. However, downregulating Nrf2 using siRNA attenuated all of these effects in a significant manner [53]. Similar results were replicated in an in vivo study using HFD to impair endothelial function by increasing vascular oxidative stress. When the mice were treated with resveratrol, the oxidative stress induced by HFD was attenuated and the protective effects were diminished when HFD-fed *Nrf2 −/−* mice were given the same resveratrol treatment [53]. In HepG2 cells, resveratrol activated the ERK pathway significantly but not the p38 and JNK pathways, which lead to an increase in Nrf2 translocation into the nucleus and higher expression of HO-1. Thus, the mechanism of action for the increase in antioxidant enzymes from resveratrol treatment may in part be mediated by the ERK pathway and phosphorylation of Nrf2 [54]. 

Resveratrol is a known sirtuin activator [55] and most of the effects of resveratrol appear to be related to its ability to activate sirtuin 1 (Sirt1). When mouse renal tubular epithelial cells were exposed to cadmium (Cd), those cells experienced an increase in mitochondrial ROS production with a decrease in mitochondrial function and biogenesis. The mechanism of Cd-induced nephrotoxicity is through dramatically decreasing Sirt3 protein expression and activity, as well as promoting acetylation of forkhead box O3 (FoxO3a). Cd exposure also leads to a decreased binding affinity of FoxO3a to the promoters of both peroxisome proliferator-activated receptor-gamma coactivator 1-alpha (PGC-1α) and superoxide dismutase 2 (SOD2) which are both regulators of mitochondrial genesis and mitochondrial ROS metabolism. Resveratrol reduced mitochondrial ROS generation by promoting Sirt3 enrichment within the mitochondria and subsequently upregulating the FoxO3a-mediated mitochondria gene expression of PGC-1α and SOD2. Sirt3 is the primary mitochondria-targeted deacetylase that is predominantly expressed in highly metabolic tissues and has been shown to bind to and deacetylate several metabolic and respiratory enzymes that regulate mitochondrial ROS generation and important mitochondrial functions. Sirt3 induces FoxO3a translocation to the nucleus and augments FoxO3a dependent antioxidant defense mechanisms through upregulation of PGC-1α and SOD2. This suggests that the mechanism of action for resveratrol-attenuated Cd-induced cellular damage is, in part, mediated through activation of the Sirt3/FoxO3a signaling pathway [56]. 

### 3.4. 1-[2-cyano-3,12-dioxooleana-1,9(11)-dien-28-oyl] imidazole (CDDO-Im)

The compound 2-cyano-3,12-dioxooleana-1,9-dien-28-oic acid (CDDO) is a synthetic oleanane triterpenoid and developed from ursolic acid and oleanolic acid, while attempting to find new anti-inflammatory agents from these natural compounds [57]. The imidazolide derivative of CDDO, CDDO-Im, is reported to be more potent than its parent compound in inducing phase II detoxifying enzymes [16], causing apoptosis of cancerous cells [58,59,60], and inhibiting NF-κB [61]. In our previous study comparing CDDO-Im and CAPE, we have reported that CDDO-Im is a more potent cytoprotectant against oxidative stress generated in human endothelial cells [8]. Both CAPE and CDDO-Im affected major cellular functions in a similar fashion as evidenced by microarray analysis, however, the effect of these two compounds on canonical pathway genes indicated that CDDO-Im upregulated more relevant genes and to a higher level compared to CAPE. The possible mechanisms for CDDO-Im cytoprotection were attributed to the induction of HO-1, activation of Nrf2 signaling pathway in response to oxidative stress, and elevation of the expression of heat shock proteins [8]. A recent time-course study revealed gene expression pattern induced by CDDO-Im in human endothelial cells [13]. Genes that upregulated by this compound within an hour include early growth response gene 1, 2, and 3. Expression of genes responsible for cytoprotection against oxidative stress such as heme oxygenase-1 is increased between 3 and 6 h incubation with CDDO-Im. Gene expression after 24 h incubation is found similar to that of the control group. Further investigation through gene expression and network analysis identified key players responsible for the cytoprotection of CDDO-Im including mitogen-activated protein kinase kinase 1 (MAP2k1) and Dual specificity protein phosphatase 1 (DUSP1). MAP2K1 lies upstream of mitogen-activated protein kinases (MAPKs) and confers its regulation through many extracellular and intracellular signals [62]. The MAPKs are also known as extracellular signal-regulated kinases (ERKs), which functions as beginning points for downstream signaling transduction events [63]. MAP2K1 is triggered through the binding of extracellular ligands to cell surface receptors, which sequentially activate RAS and RAF1 [64]. This activation of RAF1 leads to phosphorylation of the tyrosine and threonine residues of MAP2K1which then leads to activation and transduction of the MAPK/ERK pathway. Another crucial element of CDDO-Im analysis by Bynum et al. [13] is the identification of the *DUSP1*gene at the 0.5 hour point. There are several anti-inflammatory drugs that also act through the *DUSP1* gene including glucocorticoids [65]. *DUSP1* is a mitogen-activated protein kinase phosphatase-1 (MKP-1) and it modulates inflammation by dephosphorylating the tyrosine and threonine residues on MAPK. These findings are consistent with other studies that *DUSP1*is mediated by upstream activation of MAP2K1 [66]. 

## 4. Role of Oxidative Stress in Disease Conditions

### 4.1. Cardiovascular Diseases

When there is an imbalance of ROS in the body, this is known to cause oxidative stress, which damages important cellular mechanisms. One system that is greatly affected by oxidative stress is the cardiovascular system. ROS are involved in many vascular diseases and can alter the functional properties of endothelial cells in the vascular wall [67]. It is well-known that oxidized low-density lipoprotein (ox-LDL) is associated with cardiovascular diseases and this mechanism has been shown to work through the activation of NF-κB and induction of ROS formation in HUVECs, suggesting that ROS is correlated with the production of ox-LDL [68]. Ox-LDL is thought to be internalized by macrophages and endothelial cells which then leads to foam cell production and endothelial dysfunction [69]. This internalization of ox-LDL and dysfunction of endothelial cells as well as foam cell production can lead to the stimulation of endothelial cell apoptosis, induction of adhesion molecules, and inhibition of vasodilator functions [70]. 

Another way ROS plays a part in cardiovascular diseases is through angiotensin II (Ang II) which has been associated with many different cardiovascular diseases such as hypercholesterolemia, left ventricular hypertrophy (LVH), hypertension, atherosclerotic coronary artery disease, diabetes and heart failure [71]. This is due to the role of Ang II as a potent vasoconstrictor which works through the activation of NAD(P)H oxidase (Nox), a producer of ROS in vascular cells. Nox is an essential enzyme to maintain a normal physiological state but when the Nox enzyme is upregulated and over reactive, it can induce overproduction of ROS and cause oxidative stress leading to cardiovascular diseases [67]. Added sugars have been linked to cardiovascular diseases and are shown to be mediated through an increase in ROS production as glucose generates ROS through different pathways such as the sorbitol pathway, insulin pathway, activated glycation, Nox, and the mitochondrial pathway. This suggests that atherosclerosis, peripheral vascular disease, coronary artery disease, heart failure, hypertension, cardiomyopathy and cardiac arrhythmias can be caused by added sugars and this effect is mediated through ROS [72]. 

Oxidation of polyunsaturated fatty acids catalyzed by lipoxygenase enzymes has also been implicated in the development of cardiovascular diseases. In abdominal aortic aneurysms and atherosclerotic plaques, there is an increased expression of 5-lipoxygenase showing more evidence of cardiovascular diseases being mediated by ROS [73]. A heme-containing peroxidase, myeloperoxidase (MPO), is expressed in monocytes and neutrophils and may contribute to lipid oxidation in atherosclerosis through ROS [2]. Protection against oxidative stress could potentially decrease the incidence of cardiovascular diseases. 

A number of studies have been conducted on natural compounds and their antioxidant effects on the cardiovascular system. A study on CAPE showed a therapeutic effect of CAPE on rats with radiation-induced injuries. Rats were exposed to 7 Gy gamma radiation and injected with CAPE (10 μmol/kg body, i.p.) thirty minutes after for seven days in a row. The gamma radiation caused a significant increase in xanthine oxidase (XO), MDA, and adenosine deaminase (ADA) activities while significantly decreasing levels of GPx, total nitrate (NO), catalase and SOD activities in the heart tissue. This was accompanied by augmented lipid fraction levels and the activities of creatine phosphokinase, LDH and AST in the serum. Irradiated rats treated early with CAPE showed a significant increase in SOD and NO level, and a significant decrease in XO, ADA and MDA level in the heart tissue. This was accompanied by an increase in serum enzymes and a restoration of serum lipid profiles and cardiac enzymes. The results suggest that CAPE could have therapeutic effects in rats with gamma irradiation-induced cardiac-oxidative impairments [74]. However, poor aqueous solubility of CAPE is a limiting factor in the therapeutic potential of CAPE and structural modifications or nanoparticle-formulations may show superior beneficial effects [75,76].

Likewise, curcumin supplementation resulted in attenuation of cardiac hypertrophy progression, preserved endothelium-dependent relaxation, and increased survival rate in a rat model of transverse aortic construction. The study suggested that curcumin’s beneficial effects on the cardiovascular system may be due to upregulation of Na^+^/Ca^2+^ exchanger (NCX). Curcumin’s effect may come from curcumin’s ability to block the AT1 receptor which then inhibits cardiac remodeling after angiotensin II infusion and leads to upregulation of NCX expression [77]. Additionally, anti-inflammatory and antioxidant effects of curcumin coupled with activation of ROS scavenging enzymes like SOD, GPx and catalase [78] and increasing nitric oxide bioavailability to improve endothelial function [79], which may contribute towards its protective effects in cardiovascular diseases. 

A study on a synthetic CDDO derivative, dihydro-CDDO-trifluoroethyl amide (dh404) showed therapeutic effects of this compound on cardiomyocytes. Dh404 caused an interruption of the Keap1-Cul3-Rbx1 E3 ligase complex-mediated Nrf2 ubiquitination by modifying Cys-151 on Keap1 which caused Nrf2 translocation. Dh404 also enhanced Nrf2 nuclear translocation, activated Nrf2 driven transcription, stabilized the Nrf2 protein and suppressed oxidative stress induced by Ang II in cardiomyocytes. Nrf2 knockdown blocked most of dh404’s antioxidative effects and dh404 activated Nrf2 signaling in the heart. Altogether, these results show that dh404 can have a therapeutic effect on cardiac diseases via the suppression of oxidative stress through Nrf2 activation [80]. 

A number of studies demonstrated that the cardioprotective mechanisms of resveratrol may be attributed its antioxidant effects. Although multiple molecular targets including Sirt-1, AMPK, Nrf2, and NF-κB, have been identified to play an important role in the health benefits of resveratrol against cardiovascular diseases, one of the key cardioprotective mechanisms is the ability of resveratrol to scavenge free-radicals [81] and increase the bioavailability of NO [82]. Upregulation of endothelial NO synthase favors improved endothelial function, NO-mediated vasodilation, decreased aggregation of platelets further decreasing the risk of atherosclerosis formation and progressions [83]. Resveratrol also demonstrated cardioprotective effects in a chronic heart failure (CHF) rat model where, supplementation with resveratrol after the surgery to permanently ligate the left coronary artery to cause CHF. Serial echocardiography revealed an improved AV-coupling, as well as, significantly improved LV systolic function. A measurement of arterial stiffness using aortic pulse wave velocity showed a significantly lower stiffness in the resveratrol enriched group versus the control. The results of this study showed that a resveratrol-enriched diet could reduce cardiovascular functional and structural deterioration in CHF [84]. 

### 4.2. Obesity and Metabolic Syndrome

Oxidative stress plays an important role in the pathogenesis of obesity and related conditions. Obesity is associated with the pathological expansion of white adipose tissue where the highly plastic adipose tissue secretes a variety of adipokines contributing to a chronic low-grade inflammation. Several studies have shown a direct relationship between adipogenesis and elevation of ROS levels [85]. Increased oxidative stress associated with adiposity was demonstrated in KKAy obese mouse models where plasma lipid peroxidation markers were significantly higher than control mice. Furthermore, the plasma levels of ROS including hydrogen peroxide were elevated significantly compared to control mice suggesting increased oxidative stress with obesity [86]. Leptin, an adipose tissue-derived hormone, activates ROS in the vascular endothelial cells and stimulates NF-κB contributing to the inflammatory response and insulin resistance [87]. High fat diet-induced obesity and systemic oxidative stress can further increase insulin resistance and antioxidants were shown to confer protection under these conditions [88]. 

While obesity is a risk factor for type 2 diabetes, the increased oxidative stress as a result of expanding adipose tissue is a major risk factor in the development of diabetes [89]. Hyperglycemia decreases the insulin binding to its receptors in the peripheral tissues like skeletal muscle and adipocytes [90,91]. Additionally, elevated ROS or exposure to hydrogen peroxide was shown to alter insulin signaling and decrease glucose uptake by peripheral tissues [92]. This reduction in glucose uptake is accompanied by an increase in hepatic glucose production placing a burden on the pancreatic β cells to secrete more insulin resulting in failure and dysfunction of β cells [93,94].

It is well established that higher intracellular glucose concentrations and excessive consumption of fat promotes oxidative stress and alterations in diet plays a significant role in the prevention of metabolic conditions. Polyphenolic compounds, due to their ability to decrease oxidative stress, were shown to be beneficial in the prevention of obesity and in enhancing glucose metabolism in human clinical trials [95]. Although a number of natural compounds were shown to exhibit anti-obesity effects, there are very few reports on the effects of CAPE on obesity. CAPE decreases oxidative stress in 3T3-L1 adipocytes through the inhibition of peroxisome proliferator-activated receptor γ (PPAR γ) [96,97]. PPARγ is a key transcription factor involved in the regulation of adipogenesis [98] and it is possible that the anti-adipogenic effects of CAPE are mediated through the inhibition of PPARγ[96]. In high fat diet fed mice, CAPE significantly decreased fat mass and the proposed mechanism for the decrease in adipogenesis was the inhibition of mitotic clonal expansion and presumably by interference with adipogenesis at an early stage. Additionally, CAPE inhibits NF-κB and Akt signaling pathways, contributing to the suppression of HFD-induced obesity [99]. In contrast to the effects of CAPE on maturing preadipocytes, when mature adipocytes were treated with CAPE, the transcriptional activity of PPARγ was significantly increased in a dose-dependent manner, which in turn increased glucose uptake by the adipocytes [99,100]. These studies suggest potential effects of CAPE on ameliorating metabolic syndrome by increasing insulin sensitivity and promoting adipose tissue remodeling [100].

Both curcumin and resveratrol are extensively studied for their beneficial effects on obesity and other chronic metabolic diseases. The antioxidant properties of these chemicals coupled with their anti-inflammatory properties make these phytochemicals effective anti-obesity agents. In HFD-induced obese mice, curcumin reduced macrophage infiltration in the adipose tissue thereby decreasing systemic inflammation and insulin resistance, the major contributing factors for the development of metabolic syndrome [101]. Curcumin supplementation also decreased lipid accumulation in liver and adipose tissue under both in vitro and in vivo conditions [102]. However, curcumin has poor bioavailability due to its rapid metabolism and elimination [103]. Despite poor bioavailability, human clinical trials with curcumin indicate that oral supplementation of curcumin was effective in decreasing oxidative stress under obese conditions [104]. To further enhance the effectiveness of curcumin and to improve the bioavailability, various approaches were used including administering curcumin with a complex with other reagents or using nanotechology to develop curcumin preparations. For example, piperine, the major bioactive component of black pepper, when concomitantly administered at a dose of 20 mg along with curcumin at a dose of 2 g in human volunteers, a 2000% increase in the bioavailability of curcumin was noticed. No adverse effects were reported that these doses for both the compounds in humans [105]. Likewise, nanoparticle encapsulated curcumin showed enhanced solubility, stability [106] and is currently being tested for its efficacy on insulin resistance in obese patients with non-alcoholic fatty liver disease [107].

**Figure 2:**
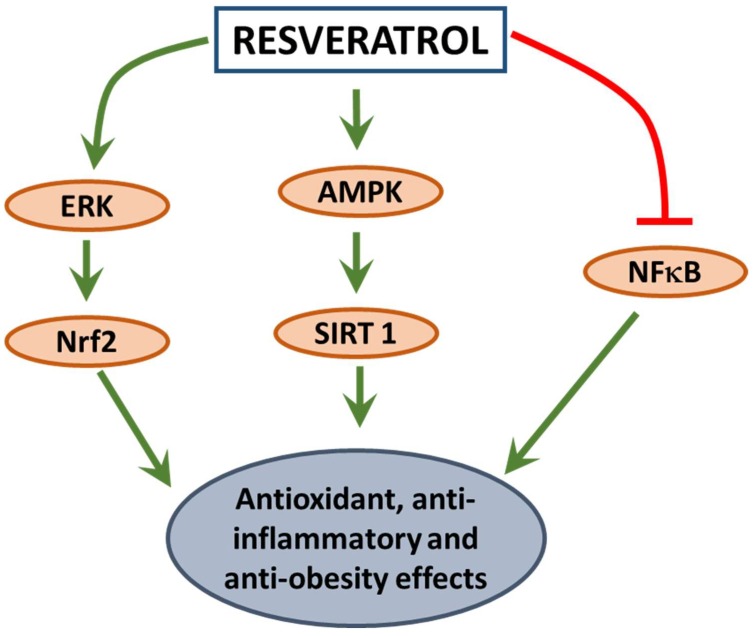
Mechanism of action of resveratrol to reduce inflammation, oxidative stress and obesity. AMPK: AMP-activated protein kinase; ERK: extracellular signal-regulated kinase; NF-κB: nuclear factor - kappa B; Nrf2: nuclear erythroid 2 - related factor; SIRT1: sirtuin 1.

Resveratrol also decreased adiposity and markers of inflammation in rodent and primate models [108,109]. The antioxidant effects of resveratrol were reported to be partly mediated through the reduction of Sirt1 and manganese superoxide dismutase levels in diet-induced obese mice [110]. Like curcumin, a number of human clinical trials examined the beneficial effects of resveratrol on various disease conditions, however studies examining the effects on obesity are limited. Although few reports indicated that resveratrol supplementation increased energy expenditure, decreased serum markers of inflammation and decreased adipocyte size in obese men [111,112], divergent effects of resveratrol were reported between the trials in metabolically challenged individuals [113]. Despite strong in vitro data demonstrating the anti-oxidant effects of these phytochemicals, further research is required to recommend these chemicals for therapeutic use in humans for chronic metabolic diseases. The mechanism of resveratrol to exert its antioxidant, anti-inflammatory, and anti-obesity effects is illustrated in Figure 2. Like curcumin, resveratrol also has very low bioavailability owing to its poor stability especially in humans. Rapid sulfate conjugation and glucuronidation are believed to be the factors contributing to the poor bioavailability of this compound [114]. To enhance the bioavailability, alternative routes of administration have been suggested to avoid the first-pass metabolism. Inhalable spray-dried formulation [115], oral transmucosal administration [116], and complexing with cyclodextrin-based nanosponges [116] have been investigated in an attempt to increase the stability, solubility and bioavailability of resveratrol. Additionally, resveratrol-loaded nanoparticles improved oxidative stress by increasing the efficiency of resveratrol to stabilize Nrf2 in A549 cells [117]. Although the use of nanotechnology to deliver phytochemicals like resveratrol and curcumin may provide advantages for chronic conditions like cancer [118], very few human clinical trials have been conducted to test the efficacy and safety of these formulations and furthermore, use of this technology for obesity and metabolic syndrome warrants additional research.

The therapeutic effects of CDDO-Im are primarily mediated through the upregulation of Nrf2, a member of the Cap’n’Collar subfamily of basic leucine zipper transcription factors, that regulates the expression of antioxidant and cytoprotective genes [119]. This is confirmed with the studies reporting that CDDO-Im supplementation prevented HFD-induced increase in adiposity and hepatic lipid accumulation in wild-type mice but failed to prevent obesogenic effects in Nrf2-disrupted mice [120]. Additional studies have shown that CDDO-Im inhibits lipogenesis in a Nrf2 dependent manner and downregulates fatty acid synthase, an enzyme that catalyzes fatty acid synthesis [120]. Thus, genes involved in carbohydrate and lipid metabolism are the major targets of CDDO-Im suggesting the potential widespread implications for this compound in metabolic diseases.

## 5. Future Perspectives

Identifying a specific drug target has become increasingly quantitative. Recently, a class of non-coding RNAs, called microRNAs (miRNAs), has gained great attention as negative regulators of gene expression that impact proliferation, differentiation, and apoptosis of cells [121]. The regulatory effects of natural compounds on miRNAs are intensively studied especially in cancer prevention and therapy [122,123]. Curcumin modulates the expression of miRNAs in various cancer cells including pancreatic cancer [124], breast cancer [125], and lung cancer [126]. Similarly, the anti-cancer effect of resveratrol through miRNA regulation is documented in prostate cancer [127], pancreatic cancer [128], and lung cancer [129]. The involvement of miRNAs in cardiovascular development and diseases was also reviewed [130], however, evidence regarding the cytoprotective effects of selected natural compounds discussed here against oxidative stress is limited. CAPE was shown to inhibit ROS- and TNFα-induced miRNA-34a in murine microglial cells [131], which may provide new insight into its mechanism of action. Resveratrol ameliorated hydrogen peroxide-induced oxidative stress through miRNA-126 activated PI_3_K/Akt pathway [132]. Clearly, further investigation to decipher the role of miRNAs in the cytoprotective effects on naturally occurring compounds is warranted. The regulation of ROS production and the Nrf2 pathway by miRNAs reviewed by Cheng et al. may provide new evidence in this direction [133]. In addition, with the invent of modern genomic tools, specific gene knockout models could potentially delineate the drug targets, however, considering the vast number of proteins involved in diseases process, generating knockout models for each target is impractical. A systems biology approach to study the mechanism of action of compounds with therapeutic potential will help explain the biological mechanisms in a way that focuses on the whole genome of an organism rather than just one gene or set of genes. This method looks at how parts of a system work together rather than how each individual part works along to elicit a response [134]. This provides a holistic and more thorough understanding of a particular phenotype and using systems biology approach may help understand the beneficial effects of natural compounds.

Within the past 60 years, drug therapy has evolved to where there is an emphasis on alleviating the side effects of non-communicable diseases. Diseases such as hypertension, asthma, ulcers, glaucoma and many types of cancers now use drug therapy. Currently, the goal for drug therapy is to find one chemical substance which has the ability to interact with just one molecular target *in vivo* in such a way that the substance can eliminate the biochemical changes associated with the disease process and bring the body’s biochemical functions back to a healthy-state. However, this is not usually the case as drugs with high-specificity have a molecular target that improves symptoms of a disease but do not resolve the central reason for the pathophysiology of that disease [135]. Due to this, although drug therapy has been successful to some extent and comes with high costs associated with development, problems related to adverse side effects still persist and long-term solutions to disease conditions are not resolved. In particular, disease conditions like cardiovascular diseases, diabetes mellitus and obesity are associated with chronic inflammation leading to dysfunction of biological mechanisms contributing to the development and progression of the disease. Since continued oxidative stress can lead to chronic inflammation, natural antioxidants surfaced as potential long-term solutions to chronic diseases. Nevertheless, the oxidation-to-antioxidation ratio is critical for maintaining normal physiological and biochemical processes and low doses of antioxidants may confer cytoprotective effects and high levels of antioxidants may disrupt the physiological balance of the system [136].

Although numerous natural compounds show promising cytoprotective and antioxidant effects as discussed in the earlier sections, large-scale randomized clinical trials conducted with antioxidants to test their protective effects on cardiovascular diseases did not result in expected results. The lack of results in the clinical trials can be partly attributed to the discrepancies in the study designs and wrong choice of antioxidants [137]. Additionally, translating in vitro doses to in vivo conditions could be challenging and a possible contributing factor for the lack of results in human studies. For example, in a phase-I clinical trial, the average peak serum concentrations of curcumin at 1–2 h after administration of 4 g and 8 g were 0.51 ± 0.11 µM and 1.77 ± 1.87 µM respectively [138]. On the contrary, a highest peak serum concertation of only 139 nM was reported after a single dose of 12 g curcumin and no curcumin was detected in the serum of subjects administered 8 g of curcumin [139]. Nevertheless, of the four natural compounds discussed in this paper, the clinical potential of curcumin has been thoroughly investigated and despite the challenges in bioavailability, difficulty in extrapolating in vitro doses to in vivo conditions and discrepancies in the results, 17 out of 49 double-blinded placebo-controlled clinical trials showed efficacy of curcumin in conditions like depression, cancer, stress, fatty liver disease, atherosclerotic risk, osteoarthritis and metabolic syndrome [140]. The degree of variability in the bioavailability of these compounds depend on a number of additional factors like the gut microflora and furthermore, pharmacogenetic research has uncovered significant differences in the population groups in metabolism and clinical effectiveness of important medications [141].

## 6. Conclusions

**Figure 3:**
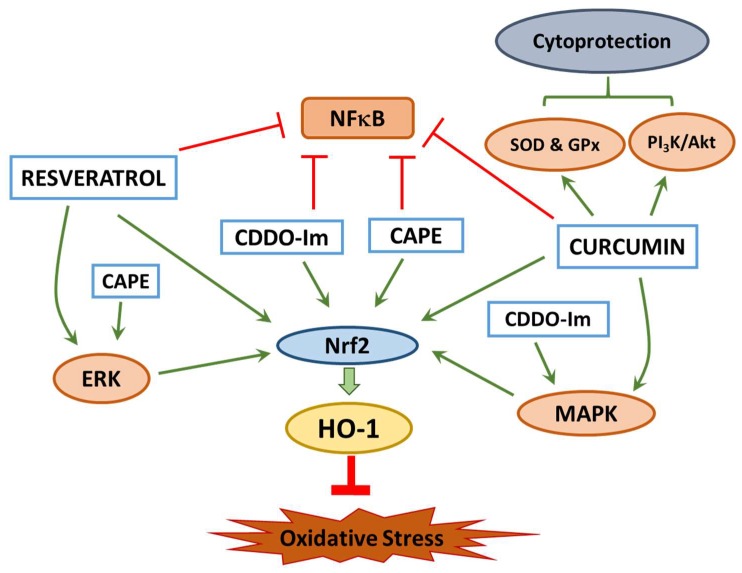
Overview of the cytoprotective mechanisms of CAPE, CDDO-Im, curcumin and resveratrol. All the four compounds inhibit NFκB [12,26,46,59] and trigger Nrf2 nuclear translocation to activate HO-1 expression. Induction of HO-1 by curcumin and CDDO-Im is mediated in part through the activation of MAPK signaling which in turn translocates Nrf2 [59]. The cytoprotective mechanism of resveratrol and CAPE include Nrf2-medaited HO-1 induction coupled with the activation of ERK signaling pathway [34,142]. CAPE: caffeic acid phenethyl ester; CDDO-Im: 1-[2-cyano-3,12-dioxooleana-1,9(11)-dien-28-oyl] imidazole; ERK: extracellular signal-regulated kinase; GPx: glutathione peroxidase; HO-1: heme oxygenase-1; MAPK: mitogen-activated protein kinase; NF-κB: nuclear factor - kappa B; Nrf2: nuclear erythroid 2 - related factor; PI_3_K/Akt: phosphatidylinositol 3-kinase/ protein kinase B; SOD: superoxide dismutase.

In conclusion, more research is needed on the mechanism of action of cytoprotective compounds to find how they work to alleviate oxidative stress. Selected natural compounds discussed in this article showed a common mechanism of action through activation of Nrf2 mediated signaling pathway and upregulation of antioxidant enzymes such as HO-1. These compounds also activate different kinases and induce phosphorylation events, which may provide an alternative mechanism of cytoprotection. Curcumin is shown to mediate PI_3_K/Akt and p38/JNK/MAPK pathways and inhibit the NF-κB pathway. Resveratrol works through the ERK pathway to induce transcription of GST, Cat, NQO1, SOD and HO-1. Like curcumin, resveratrol also modulates multiple signaling mechanisms and demonstrates an effect on the NF-κB and the sirtuin/FoxO3a pathways. The research on CDDO-Im shows that this compound upregulates HO-1 through MAP2K1 to promote the activity of other kinases which, in turn, upregulates antioxidant enzymes. Thus, a number of in vitro studies demonstrated potent effects of these compounds on reducing oxidative stress. Cytoprotective mechanism of these four natural compounds is summarized in Figure 3. However, there is a gap between the in vitro studies and animal studies/human clinical trials conducted with these compounds, owing to the difficulty in translating in vitro effects to clinical application. Systems biology approach can help gain more insight into molecular targets that we can take advantage to reduce oxidative stress. Additionally, a combination of these natural compounds to emulate multifocal signal modulation therapy for the treatment of cardiovascular diseases, obesity and diabetes can result in beneficial effects. Regardless of whether these natural compounds can effectively be used for the treatment of pathological conditions, additional studies aimed at refining the existing knowledge on the mechanism of action and molecular targets will aid in developing pharmaceuticals for therapeutic purposes.

## Figures and Tables

**Table 1:** Cytoprotective effects of selected natural compounds.

Compounds	Mechanism of Cytoprotection	References
CAPE	Induction of HO-1	Wang et al. [8], Scapagnini et al. [9]
	Inhibition of IL5 and IFNγ production	Wang et al. [10]
	Tyrosine kinase inhibitor	Patel et al. [11]
	Inhibition of NF-κB activation	Natarajan et al. [12]
CDDO-Im	Induction of heat shock protein family Activation of MAP2K1 and DUSP1 Induction of glutathione (GSH) Activation of Nrf2/ARE pathway and induction of Nrf2 downstream genes	Wang et al. [8] Bynum et al. [13] Speen et al. [14] Reisman et al. [15], Liby et al. [16]
Resveratrol	Upregulating HO-1 and activation of PI3K/Akt/Nrf2 pathway	Hui et al. [17], Jin et al. [18]
	Inhibition of caspase activation and proteolytic cleavage of tau at Asp^421^	Means et al. [19]
	Increasing SIRT1 deacetylate activity	Wang et al. [20]
	Suppression of Wnt/β-catenin signaling	Xu et al. [21], Geng et al. [22], Xie et al. [23]
Curcumin	Induction of HO-1and activation of Nrf2/ARE signaling	Motterlini et al. [24], Balogun et al. [25]
	Induction of Glutathione Biosynthesis, Inhibition of NF-κB Activation and Interleukin-8 Release	Biswas et al. [26]
	Suppression of STAT-3 and Wnt/β-catenin and activation of PPAR-γ	Shehzad et al. [27]
